# Phage Display Identification of CD100 in Human Atherosclerotic Plaque Macrophages and Foam Cells

**DOI:** 10.1371/journal.pone.0075772

**Published:** 2013-09-30

**Authors:** Maria Carolina Aquino Luque, Paulo Sampaio Gutierrez, Victor Debbas, Waleska Kerllen Martins, Pedro Puech-Leao, Georgia Porto, Verônica Coelho, Laurence Boumsell, Jorge Kalil, Beatriz Stolf

**Affiliations:** 1 Heart Institute of São Paulo (InCor), HC-FMUSP, São Paulo, São Paulo, Brazil; 2 Clinical Immunology and Allergy, Department of Clinical Medicine, University of São Paulo Medical School - HC-FMUSP, São Paulo, São Paulo, Brazil; 3 Institute for Investigation in Immunology - INCT - National Institute of Science and Technology, São Paulo, São Paulo, Brazil; 4 Inserm U567, Institut Cochin, University Paris Descartes, Paris, France; 5 Department of Biochemistry, Institute of Chemistry, University of São Paulo, São Paulo, Brazil; 6 Department of Parasitology, Institute of Biomedical Sciences, University of São Paulo, São Paulo, Brazil; University of Milan, Italy

## Abstract

Atherosclerosis is a complex disease in which vessels develop plaques comprising dysfunctional endothelium, monocyte derived lipid laden foam cells and activated lymphocytes. Considering that humans and animal models of the disease develop quite distinct plaques, we used human plaques to search for proteins that could be used as markers of human atheromas. Phage display peptide libraries were probed to fresh human carotid plaques, and a bound phage homologous to plexin B1, a high affinity receptor for CD100, was identified. CD100 is a member of the semaphorin family expressed by most hematopoietic cells and particularly by activated T cells. CD100 expression was analyzed in human plaques and normal samples. CD100 mRNA and protein were analyzed in cultured monocytes, macrophages and foam cells. The effects of CD100 in oxLDL-induced foam cell formation and in CD36 mRNA abundance were evaluated. Human atherosclerotic plaques showed strong labeling of CD100/SEMA4D. CD100 expression was further demonstrated in peripheral blood monocytes and in *in vitro* differentiated macrophages and foam cells, with diminished CD100 transcript along the differentiation of these cells. Incubation of macrophages with CD100 led to a reduction in oxLDL-induced foam cell formation probably through a decrease of CD36 expression, suggesting for the first time an atheroprotective role for CD100 in the human disease. Given its differential expression in the numerous foam cells and macrophages of the plaques and its capacity to decrease oxLDL engulfment by macrophages we propose that CD100 may have a role in atherosclerotic plaque development, and may possibly be employed in targeted treatments of these atheromas.

## Introduction

Atherosclerosis is one of the most prevalent diseases worldwide. Atherosclerotic lesions are present as asymmetrical focal thickenings of the intima, the innermost layer of the artery, and result from focal accumulation of blood-borne inflammatory and immune cells, vascular endothelial cells, extracellular matrix, lipids and acellular lipid-rich debris [1].

Macrophages and foam cells are the most abundant cell type in the atheromas although T cells are also present in a significant number especially at the early stages [2]. Foam cells differentiate from activated macrophages that express scavenger receptors (SR) which uptake cholesteryl esters that accumulate in the cytosol [3]. Scavenger receptors clear residual components and are additionally involved in lipid metabolism, binding modified low-density lipoproteins (LDL) [4]. Unlike the native LDL receptor, SR such as CD36 are not down-regulated by increases of intracellular cholesterol levels, resulting in a continued uptake of modified LDL and foam cell formation [5].

Identifying markers of atherosclerotic plaques may be very useful to improve diagnosis and treatment. In fact, diagnosis of the disease usually occurs in an advanced stage (vulnerable plaques), and treatment is quite invasive. The human vulnerable plaque is one of the toughest cases in model design and none of the available animal models seem to precisely reproduce it [6]. Considering this, we decided to search for markers of human fresh carotid plaques using phage display peptide libraries, which are efficient tools for the identification of markers for several tissues and cell types [7–10]. Among the phages that bound to two different human plaques after subtraction using two different normal carotids we found sequences homologous to plexins and semaphorins. One of them was homologous to plexin B1, a high affinity receptor for the semaphorin CD100.

Semaphorins are secreted and membrane-associated proteins characterized by a conserved amino-terminal SEMA domain, and are divided in eight subclasses [11]. CD100/SEMA4D belongs to the class 4 of semaphorins, and is the only for which membrane and soluble forms endow distinct functional properties [12]. It is expressed on the majority of hematopoietic cells (B, T, NK and myeloid cells), and expression generally increases after cell activation [12]. T lymphocytes express the highest levels of CD100 [13,14], followed by platelets [15,16] and monocytes [17], all of which can be found in human plaques. Tumor associated macrophages also express CD100, being associated with tumor vascularization, growth and metastases [18]. Human CD100 was shown to influence migration and cytokine production in monocytes [19], and is involved in T cell activation and B cell survival. Monocytes express higher levels of CD100 when stimulated with IFN-γ, a central cytokine in atherosclerosis [1,13].

Differently from humans, murine CD100 couples to CD72 expressed in B lymphocytes and APCs, participating in the interactions between these cells and shutting off negative restraining signs in B cells [12], thus modulating humoral responses [12,20]. A few studies have been performed to establish a possible role for CD100 in the development of atherosclerosis in the mice model; they have shown that lack of Sema4D reduces platelet hyperactivity otherwise found in dyslipidemia, thus conferring protection against atherosclerosis [15]. Moreover Sema4D^-/-^ApoE^-/-^ mice presented decreased lipid staining, macrophage infiltration and intimal neovascularization in the aortic plaques, suggesting that Sema4D facilitates plaque growth by promoting neovascularization through induction of endothelial cell migration [21]. Nonetheless, there are still no reports about CD100 expression in human atherosclerotic plaques. Furthermore, there is no evidence of CD100 expression in cultured or tissue human macrophages besides the ones found in the tumoral environment.

Considering the binding of CD100 high affinity receptor to plaques revealed by phage display and the molecule’s angiogenic and immunological roles, we sought to evaluate CD100 expression in human atherosclerotic lesions. Immunohistochemical analysis indicated strong CD100 expression in macrophages and foam cells, as well as in cultured macrophages and in *in vitro* differentiated foam cells.

Peripheral blood (PB) monocytes stimulated with IFN-γ presented augmented CD100 transcripts, and CD100 RNA abundance was inversely correlated to monocyte-macrophage-foam cell differentiation in PB derived cells. Contrarily to the pro-atherogenic effect of CD100 described in mice models [21], we observed a possible anti-atherogenic effect of soluble CD100 by the reduction of oxLDL-induced foam cell formation, presumably by the observed decrease in CD36 expression.

## Methods

### Ethics Statement

Informed written consent was obtained from each patient/family before collection of samples and the study protocol conforms to the ethical guidelines of the Hospital das Clínicas (HC - University of São Paulo School of Medicine) Human Research Committee.

### Human specimen collection

Four normal carotid samples were obtained from donors of the Organ Transplantation of the Hospital das Clínicas of the São Paulo School of Medicine (controls with no vascular disease). Researchers involved in the project were present during heart removal from the donor and received a fresh fragment of approximately 5cm of the carotid that would not be used for transplantation. The fragment was divided in two pieces: one was transferred to 10% formalin for paraffin embedding and the other was kept in DME medium for phage display subtraction procedure. The family of the donors had previously signed an authorization form specific for this project donating the artery fragment to the “Laboratory of Immunology, Heart Institute”. Twenty seven carotid plaques were obtained from endarterectomies at the Heart Institute of São Paulo. Patients with infectious diseases, cancer, malignant arterial hypertension, previous surgical intervention in the same local, previously transplanted or immunosuppressed were excluded. Carotid plaques used for panning were classified as AHA 5B/C (plaque with calcification and low lipid levels) and 5A (plaque with fibrous cap) (Stary et al., 1994 [22]; Stary et al., 1995 [23]). The plaque shown in immunohistochemistry/immunofluorescence experiments belongs to the same AHA 5A classification. Paraffin sections of a coronary plaque and a normal coronary artery were gently provided by Dr Lourdes Higuchi. Coronary fragments were collected post-mortem (autopsies) of patients who died of acute myocardial infarction at the Heart Institute, Incor - Hospital of the Faculty of Medicine, University of São Paulo, between 1985 and 1999. At the time of death the family signed a consent form authorizing the autopsy pathology service in the same institution. This authorization form allowed the use of this material for further research. Collection of both samples (carotid and coronary arteries) was approved by the Heart Institute Scientific Committee and CAPPesq (Hospital das Clinicas Ethics Committee).

### Phage Display

The phage peptide library CX7C used in this project was produced by Dr. Wadih Arap, Dr. Renata Pasqualini (MD Anderson Cancer Center, USA) and Dr. Erkki Koivunen (Helsinke University, Finland). The pre-clearing (subtraction) step was done in a normal fresh carotid artery pre-washed in DME medium and blocked with DME with 3% BSA for 15 minutes in ice. 10^9^ TU library phages in 100 µL of DME with 3% BSA were incubated with the luminal face of the normal carotid for 30 minutes. Unbound phages were recovered and incubated for 2 hours with the luminal face of a fresh carotid plaque previously blocked with DME containing 3% BSA. The plaque was carefully washed with DME and bound phages were recovered by infection with *E. coli* K91kan, amplified and precipitated using PEG/NaCl. These two steps were repeated using a different normal carotid for pre-clearing and another plaque for panning. After the two cycles of pre-clearing and panning (selection), 100 phages were randomly selected and sequenced. Peptide sequences were translated using Expasy, aligned using ClustalW and compared to known sequences using BLASTp.

### Cell culture

Peripheral blood (PB) mononuclear cells were obtained by density gradient centrifugation with Ficoll (Invitrogen, Carlsbad, CA) and human monocytes were purified by positive selection using anti-CD14 magnetic beads (Miltenyi Biotec, Germany). Purity of monocytes was above 97% as verified by flow cytometry analysis. Human T lymphocytes were purified by negative selection using Pan T Isolation kit (Miltenyi Biotec, Germany). PB monocytes macrophages and foam cells were cultured in RPMI-1640 (Gibco, NY) containing 10% (v/v) calf serum (Hy Clone, Utah), 10mM HEPES, 1mM glutamine, 200U/mL penicillin, 2mg/mL streptomycin and 1mM sodium pyruvate. T lymphocytes were cultured in DMEM (Gibco, NY) with 10% (v/v) calf serum (Hy Clone, Utah), 10mM HEPES, 1mM glutamine, 200U/mL penicillin, 2mg/mL streptomycin, 1mM sodium pyruvate and IL-2, IL-7 e IL-15 (R&D, Minneapolis, MN) 40U/mL each.

PB monocytes were differentiated to macrophages with 20ng/mL Macrophage Colony Stimulating Factor (M-CSF) (Peprotech, Rocky Hill, NJ) for 7 days. Macrophages were differentiated into foam cells using a protocol adapted from Banka et al., 1991 [24], by incubation with oxLDL (50μg/ml) for 48h. oxLDL was gently provided by Dr Claudia Andrade from the Vascular Biology Laboratory, Heart Institute (InCor), São Paulo, Brazil. LDL was isolated from whole blood of healthy volunteers and oxidized as described by Cominacini et al., 2000 [25]. Briefly, whole blood was collected into tubes containing EDTA (1 mg/ml), centrifuged at 2000 rpm for 20 min at 4°C and processed for LDL separation by sequential flotation in NaBr solution containing 1 mg/ml EDTA. LDL was separated by gel filtration on PD-10 Sephadex G-25M gel in 10 mM phosphate-buffered saline. OxLDL was obtained by exposure to 5 mM CuSO4 for 18 h at 37°C. The extent of LDL oxidation procedure was determined by evaluating the level of thiobarbituric acid-reactive substances.

For stimulation, monocytes, macrophages and foam cells were seeded at 1.5x10^6^/1.5mL/well in 6-well plates and T lymphocytes at 2x10^6^/1.0mL/well in 24-well plates (Costar, Cambridge, MA). Monocytes, macrophages and foam cells were stimulated with IFN-γ 100U/mL (Peprotech, Rocky Hill, NJ) for 3h. T lymphocytes were activated CD3/CD28 beads (MACS, Myilteni Biotec, Germany) 0.625µL/mL for 24h.

### Immunohistochemistry

Tissue sections were subjected to antigen recovery with Tris-EDTA buffer in Pascal pan and blocked with Protein Block (DAKO, Glostrup, Denmark). After incubation with the primary antibodies anti-CD100 1:1400 (BD Transduction, San Jose, CA) or anti-CD68 1:300 (Santa Cruz, Santa Cruz, CA) in PBS 1% (w/v) BSA for 18h at 4°C, the samples were submitted to LSAB (DAKO, Glostrup, Denmark) procedure (biotin for 1h and streptavidin for 30min, both at 37°C). They were next incubated with DAB (DAKO, Glostrup, Denmark) for 1min30s and in Scott water for 30s, dehydrated through passages in alcohol and xylol and mounted with Entellan (Merck, Darmstadt, Germany). Positive controls were done using spleen sections in the same conditions mentioned above, and negative controls were performed using the same carotid/ coronary normal arteries and plaque sections and only LSAB (secondary antibody). CD100 labeling was also performed in thyroid tissue, used as negative control tissue. The digitalization of the images was made with the program AxioVision (Carl Zeiss Inc., Jena, Germany).

### Immunofluorescence

Tissue sections and cultured cells were blocked for 1h at room temperature with Protein Block (DAKO, Glostrup, Denmark) or with PBS 1% BSA and incubated with the primary antibodies anti-SEMA4D (clone 3B4, Abnova, Taipei, Taiwan) and anti-CD68 (mouse anti-human: clones PG-M1 and KP1, DAKO, Glostrup, Denmark; rabbit anti-human: clone H-255, Santa Cruz, CA) in PBS 1% (w/v) BSA at 4°C for 18h. After washing, slides or coverslips were incubated with the secondary antibodies for 90min at 4°C: Alexa Fluor 660 goat anti-mouse IgG (Invitrogen, Carlsbad, CA), FITC anti-rabbit IgG (Vector, Burlingame, CA) or Alexa Fluor 433 goat anti-rabbit IgG (Invitrogen, Carlsbad, CA). For cultured cells, culture medium was aspirated, and coverslips were washed three times with PBS. Cells were fixed with 2% paraformaldehyde for 30 min at RT or for 15 min at 37 °C. After washing steps with PBS the cells were permeabilized in PBS 0.1% Nonidet P40 for 30 min at RT or 15 minutes at 37 °C. After blocking, coverslips were then incubated with the primary and secondary antibodies as described above. Glass slides and coverslips were mounted with DAPI (Sigma, St. Louis, MO) (10µg/mL in glycerol: PBS 1:1) and analyzed in the Zeiss LSM 510 confocal system Meta/UV and in the inverted motorized microscope Zeiss - Axiovert 200.

### Quantitative Reverse-Transcription Polymerase Chain Reaction

Cultured cells were lysed with Trizol (Invitrogen, Carlsbad, CA) and total RNA was extracted as described by the manufacturer. 2μg of RNA were reverse transcribed to cDNA using random primers and oligodT and Superscript II reverse transcriptase (Invitrogen, Carlsbad, CA). Relative mRNA levels were assessed by quantitative reverse-transcription polymerase chain reaction with SyBR Green master mix (Applied Biosystems, Foster City, CA) in triplicates using an ABI Prism 7700 (Applied Biosystems, Foster City, CA). Primers for CD100, GAPDH, IL-1 β and STAT-1 (see below) were designed using primerexpress software and purchased from Invitrogen (Carlsbad, CA). Results were normalized using GAPDH.

Primers:
CD100(F): 5’ CGAGAAGCAGCATGAGGTGTATTG 3’
CD100(R): 5’ CGGATGTAGTTGAGGCACTCTGTC 3’
STAT1(F): 5’ CAAGGTGGCAGGATGTCTCAG 3’
STAT1(R): 5’ TTCCATGGGAAAACTGTCATCAT 3’
GAPDH(F): 5’ TGGTCTCCTCTGACTTCAACA 3’
GAPDH(R): 5’ AGCCAAATTCGTTGTCATACC 3’

### Western blotting

Cultured cells were washed with PBS and lysed with lysis buffer (1% Nonidet P-40 (v/v), 50mM Tris (pH 7.4), 150mM NaCl, 1mM EDTA, 1mM PMSF, 50 U/mL aprotinin and 1mM leupeptin) for 30 min at 4°C. After centrifugation at 12000g to remove cellular debris, the protein was quantified with BCA (Pierce, Rockford, IL). 30μg of soluble protein was separated by SDS-PAGE and electrotransferred to nitrocellulose membranes (Amersham, Paris, France). The membranes were blocked and incubated with the primary antibody anti-CD100 (BD Transduction, San Jose, CA), overnight at 4°C. After washing, membranes were incubated with anti-mouse HRP-conjugated secondary antibody for 1h at room temperature. Blots were developed using chemiluminescence detection system ECL (Amersham, Paris, France) followed by autoradiography on x-ray film (Kodak, Cedex, France) and scanning densitometry. The same blots were incubated with anti-β-actin mouse anti-human antibody (Sigma, St. Louis, MO) and developed as described.

### Measurement of oxidized LDL incorporation

*In vitro* differentiated macrophages were incubated with 50μg/ml of oxLDL for differentiation into foam cells with or without 10µg/ml of CD100 (Abnova, Taipei, Taiwan) for 48h (as suggested by Dr Boumsell). The medium was removed and the cells fixed with 10% formalin for 5 min at RT. Cells were then washed with 60% isopropanol, allowed to dry, and stained with Oil Red (Sigma O-0625, St. Louis, MO) for 10min. The wells were repeatedly washed with distilled water, and Oil Red was eluted with 100% isopropanol for 10min. The optical density (OD) was measured at 500 ηm, 0.5sec. 100% isopropanol was used as blank.

### Statistics

Kolmogorov-Smirnov test was applied to verify if the group samples presented normal distribution. In case of a Gaussian distribution, parametric paired T-student test (two groups) or ANOVA (more groups) were applied. Otherwise non-parametric Wilcoxon (two groups) or Friedman (more groups) tests were used. Correlation analyses between CD100 or STAT-1 expression along cell differentiation were performed using Spearman’s (non-parametric test) or Pearson’s coefficient (parametric test). P-values of 5% were considered significant.

## Results

### Phage display and plexin B1 phage

After two cycles of pre-clearing (subtraction) in normal carotids and selection (panning) in atherosclerotic plaques, we randomly selected and sequenced 100 phages among the ones that bound to the plaques. All 100 phages were different and alignment using ClustalW did not show important common sequences or domains.

Comparison to public databases using BLASTp indicated that one of the phages (phage B6, and only this phage) presented four consecutive amino acids (ELGC) in nine (CGFSG**ELGC**) identical to plexin B1, rendering an alignment score of 16.3. Since plexin B1 is a high affinity receptor for CD100, we aimed to validate this finding by analyzing CD100 expression in atherosclerotic plaques.

### Expression of CD100 in atherosclerotic plaques, spleen and cultured cells

**Figure 1:**
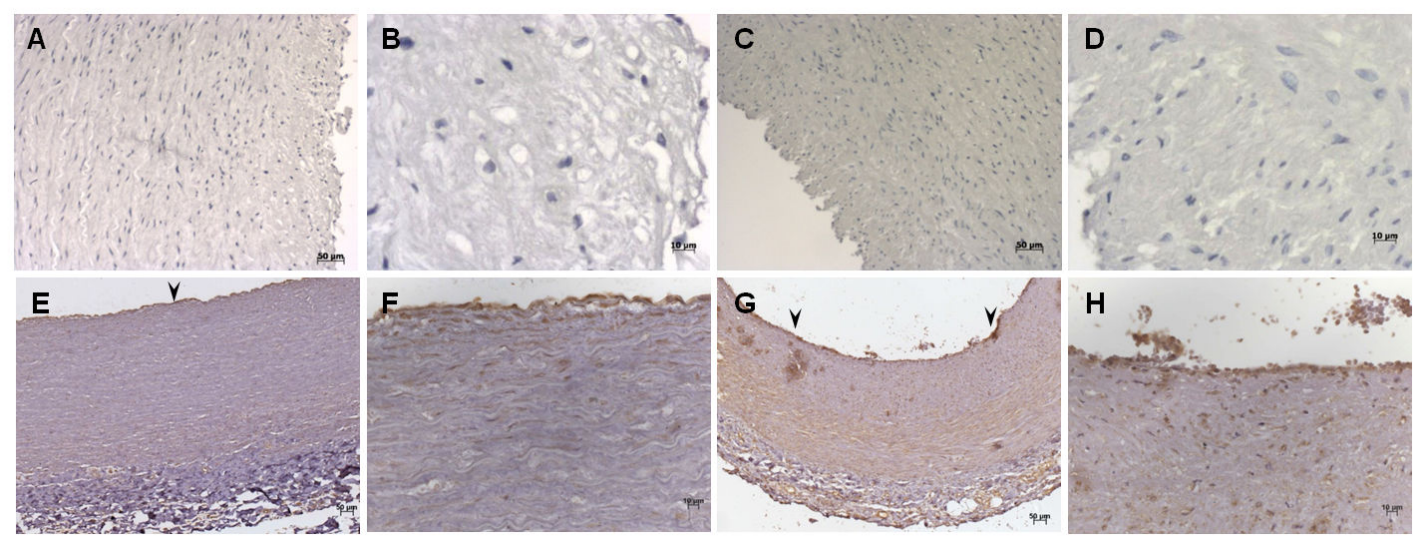
CD100 is expressed only in endothelia of normal coronary and carotid arteries. CD100 labeling (E-H) and negative controls (A-D) in normal carotid (A, B, E, F) and coronary (C, D, G, H) artery tissue sections by immunohistochemistry. Positive CD100 staining was observed only in endothelia of normal arteries (arrowheads). Scale bars: 10µm (*A*, *E*, *C* and *G*) and 50µm (*B*, *F*, *D* and *H*).

**Figure 2:**
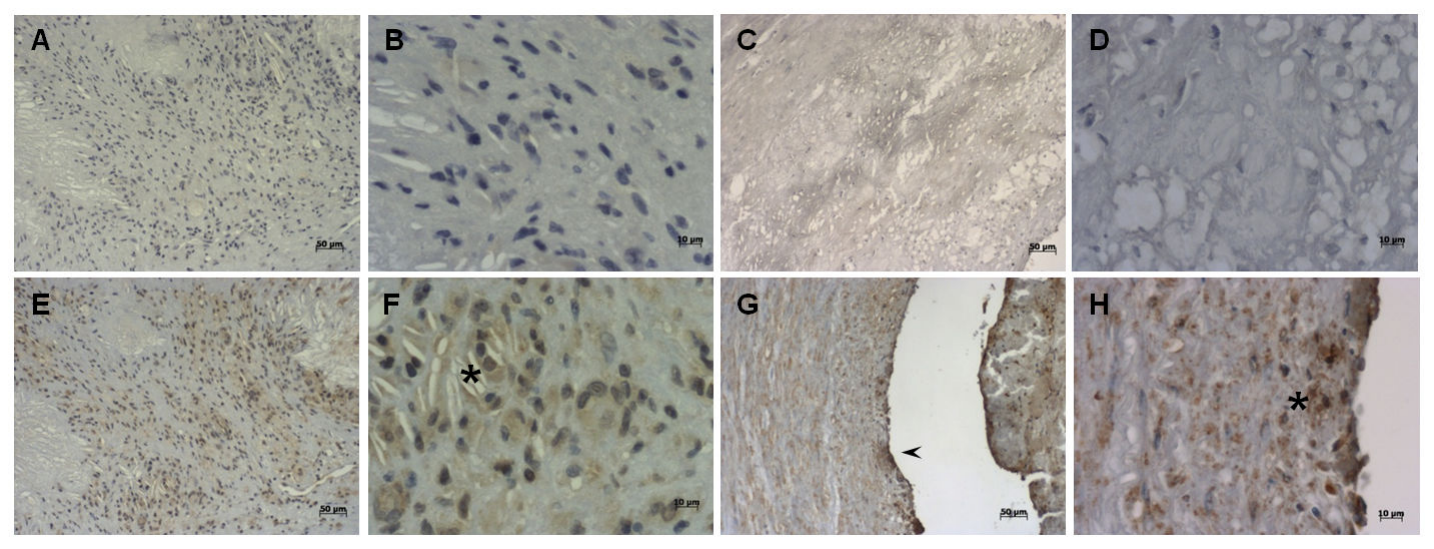
Infiltrating cells of the intima in coronary and carotid plaques are CD100 positive. CD100 labeling (E-H) and negative controls (A-D) in artery tissue sections of carotid plaques (A, B, E, F) and coronary plaques (C, D, G, H) by immunohistochemistry. Positive CD100 staining was observed only in preserved endothelium (arrowhead), and in infiltrating cells in the intima (asterisks). Scale bars: 10µm (*A*, *E*, *C* and *G*), and 50µm (*B*, *F*, *D* and *H*).

**Figure 3:**
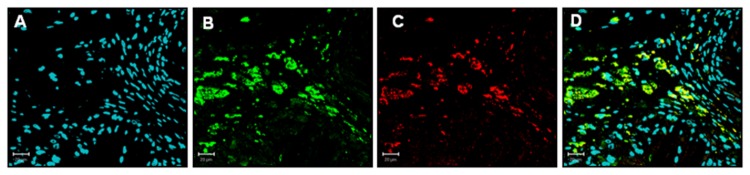
Macrophages and foam cells from atherosclerotic plaques express CD100. Immunofluorescence of carotid plaques with blue nuclei in DAPI (A), CD68 staining in FITC (*green*, B), CD100 staining in Alexa 660 (*red*, C) and co-localization of markers (D). Scale bars: 20µm.

CD100 expression was investigated in tissue sections from normal human carotid (Figure 1E, F) and coronary (Figure 1G, H) arteries and the respective carotid (Figure 2E, F) and coronary (Figure 2G, H) plaques. In most plaques analyzed there were practically no T cells, but abundant macrophages and foam cells. We observed positive cytoplasmic staining for CD100 in the vast majority of the cells in the area of the plaques occupied by foam cells (Figure 2E-H). In both normal carotid and coronary samples analyzed CD100 was stained mainly in endothelial cells (Figure 2E-H). Double staining immunofluorescence using anti-CD68 and CD100 confirmed that CD100 positively stained carotid plaque macrophages (Figure 3).

We subsequently analyzed whether conventional, tissue-resident macrophages also expressed CD100. Double staining immunofluorescence experiments in human spleen tissue sections for both CD100 and CD68 showed a great number of double positive cells (Figure S1), which confirmed that most spleen macrophages (CD68 positive) also expressed CD100. However, single positive populations for each marker could also be observed.

**Figure 4:**
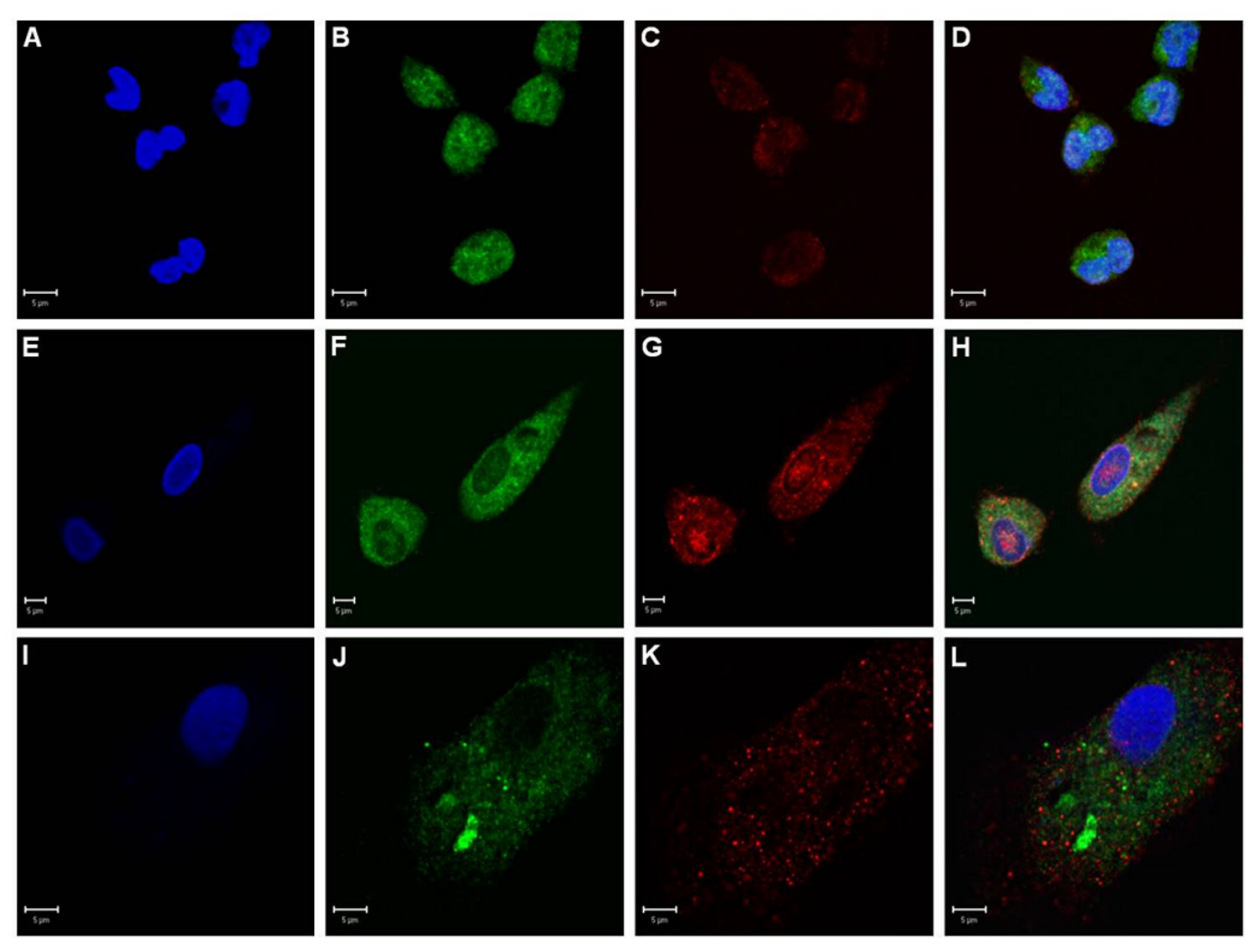
*In vitro* cultured monocytes, macrophages and foam cells express CD100. Immunofluorescence of PB monocytes (A-D), macrophages (E-H) and foam cells (I-L) showing blue nuclei in DAPI (A, E, I), CD68 (*green*; B, F, J), CD100 (*red*; C, G, K) and co-localization of all markers in D, H and L. Scale bars: 5µm.

Next we analyzed CD100 expression in cultured monocytes from peripheral blood and *in vitro* differentiated macrophages and foam cells. Double staining immunofluorescence using antibodies for CD68 and CD100 showed expression and co-localization of both markers in all PB derived cell types (Figure 4A-L)

### CD100 mRNA in cultured cells

**Figure 5:**
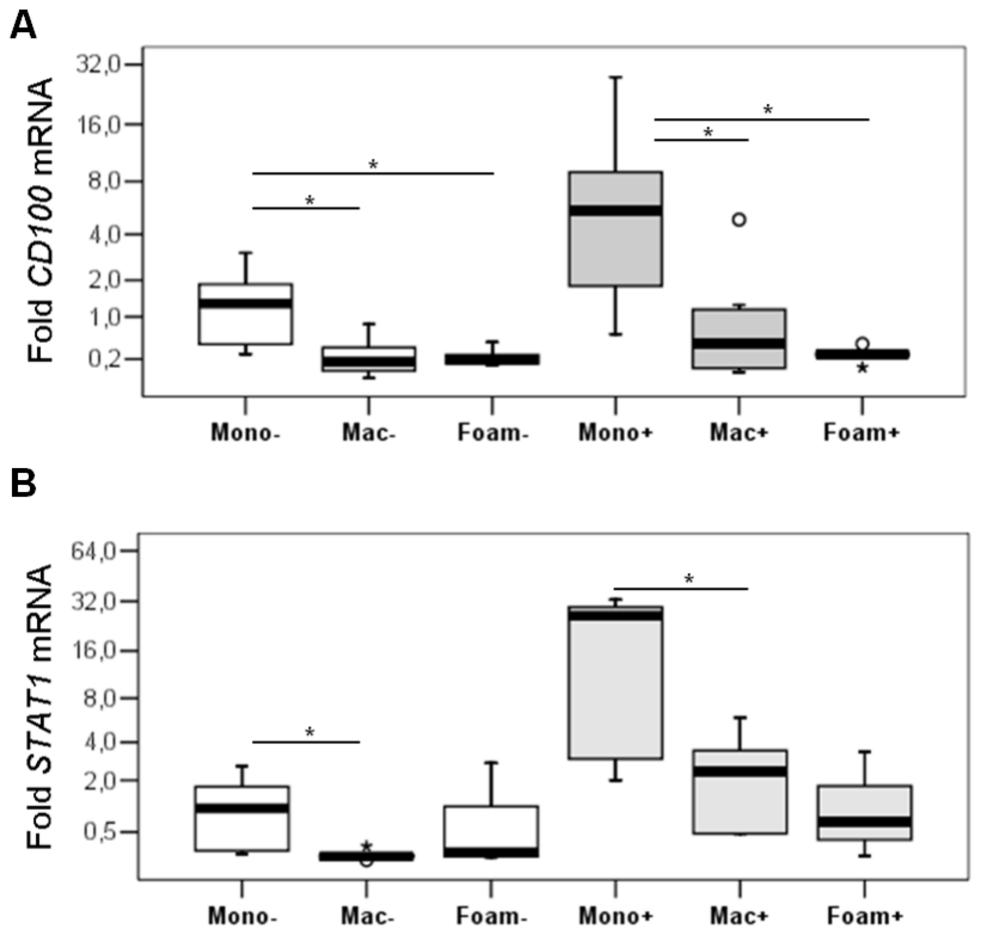
Resting and IFN-γ stimulated monocytes show increased levels of CD100 mRNA compared to macrophages and foam cells (A, B). Quantitative RT-PCR (qRT-PCR) for CD100 (A) or STAT-1 (B) in PB monocytes, macrophages and foam cells stimulated (+) or not (-) with IFN-γ. Folds relative to non stimulated monocytes (mono-), normalized with GAPDH. (a) * = significant *p* values (p≤0.05) in the comparisons in unstimulated and stimulated conditions.

We analyzed CD100 mRNA abundance using quantitative RT-PCR in cultured monocytes, macrophages and foam cells, and the effect of 24h activation with IFN-γ. Figure 5A shows that monocytes express significantly more CD100 than macrophages and foam cells (p values 0.018 and 0.028, respectively), and this difference is still observed after activation (p = 0.018). On the other hand, macrophages and foam cells have similar transcript levels both in activated and non-activated conditions. CD100 expression was inversely and significantly (p ≤ 0.007) correlated with monocyte-macrophage-foam cell differentiation with coefficients (rho) of -0.57 and -0.68 for the non-activated and activated states, respectively. Concerning activation, the only cell type that showed significant increase (p = 0.018) in CD100 after IFN-γ treatment were monocytes (Figure 5A). STAT-1 expression was assessed to confirm activation by IFN-γ [26], and we observed a significant higher expression in monocytes compared to macrophages (p = 0.028) in non-activated and activated conditions. Increased STAT-1 expression confirmed IFN-γ activation in monocytes and in macrophages (p = 0.018 and 0.028, respectively) (Figure 5B). Differently from what was observed for CD100, there was a negative correlation (rho = -0.71 with a p = 0.002) in the pattern of expression of STAT-1 along differentiation only in the activated state but not in non-stimulated cells (rho = -0.2 with a p = 0.426). The most activated cells according to STAT-1 expression were also the ones that expressed the highest levels of CD100.

### CD100 protein in cultured cells

We then compared CD100 protein abundance in lysates of PB monocytes, macrophages and foam cells, that would represent a sum of intracellular and membrane CD100, not including the soluble form. T lymphocytes stimulated or not with CD3/CD28 beads were used as positive control.

**Figure 6:**
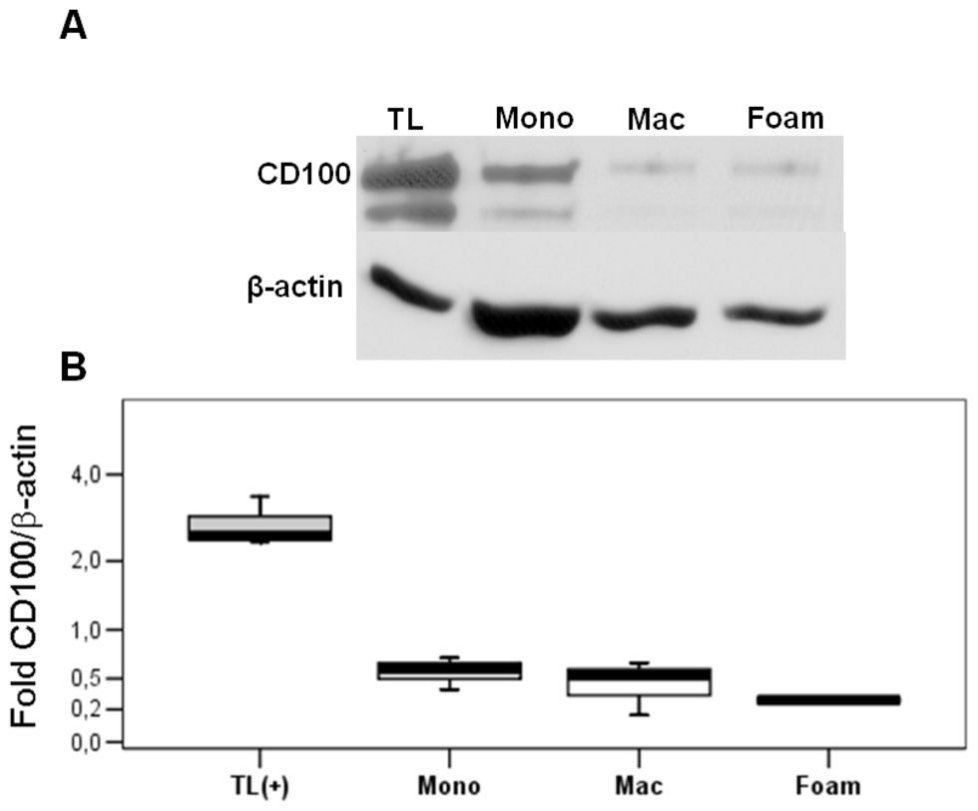
Cultured monocytes express higher amounts of CD100 protein than *in vitro* differentiated macrophages and foam cells. CD100 and β-actin protein expression was evaluated in activated T lymphocytes (TL – positive control) and PB monocytes (Mono), macrophages (Mac) and foam cells (Foam) A. Western blot showing CD100 and β-actin bands B. Densitometry of CD100 (sum of 120 and 150kDa bands) and β-actin protein bands, showing CD100/β-actin. Mean ± SD of 3 independent experiments.

In Jukart cells and other cell types, mCD100 migrates as a 150kDa monomeric form and the soluble form (sCD100) as 120kDa form, under reducing conditions [27]. Accordingly, we detected two bands of approximately 150kDa and 120kDa in PB derived cells (Figure 6A). We observed statistically similar levels of CD100 in monocytes, macrophages and foam cells (Figure 6B). In fact, there was no significant negative correlation between CD100 protein expression and differentiation (r = -0.617, with a p = 0.077). Unfortunately, the low yield of monocytes, macrophages and foam cells did not render sufficient protein for both non-activated and IFN-γ activated conditions.

### CD100 decreases LDLox uptake by macrophages and foam cell formation

**Figure 7:**
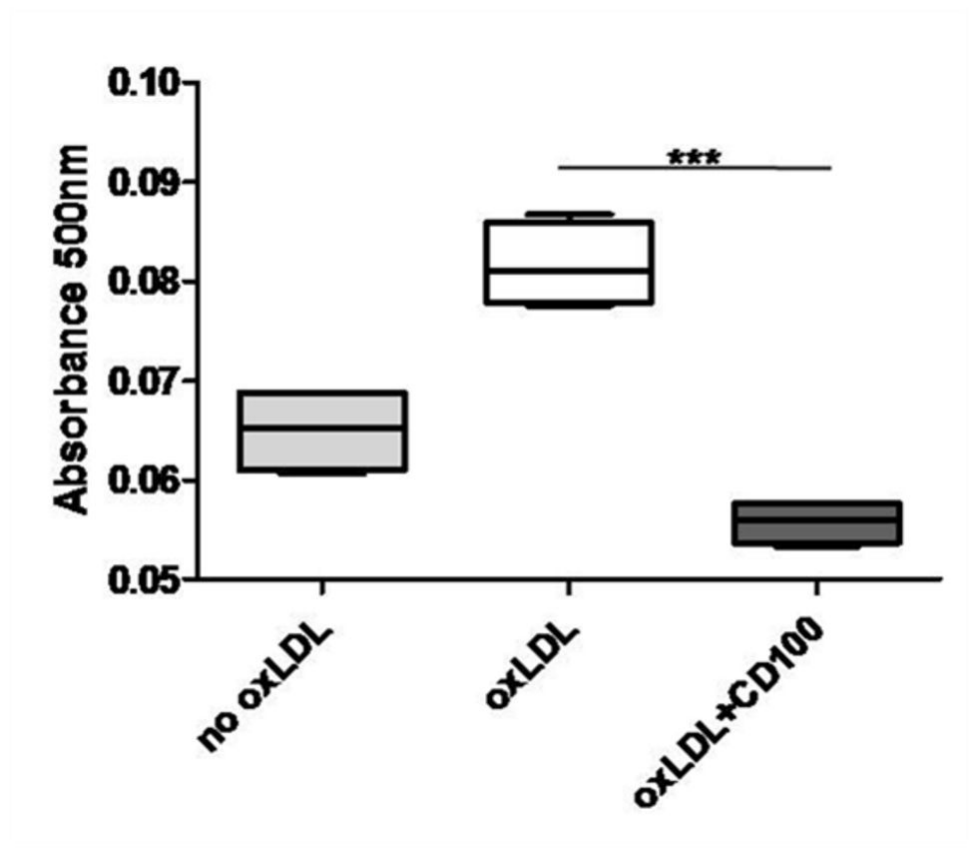
CD100 protein reduces oxLDL incorporation in macrophages. Optical density indicating incorporation of oxLDL by PB macrophages incubated or not with oxLDL or oxLDL and CD100. Mean ± SD of 5 independent experiments (*** p≤0.001).

Although CD100 expression was verified in plaque and cultured macrophages as well as its upregulation under pro-inflammatory stimuli, the effects of macrophage and foam cell-derived CD100 remained unclear. To verify whether soluble CD100 shed by these cells would have any effect on the uptake of oxLDL by macrophages, macrophages were differentiated into foam cells in the presence or not of recombinant CD100 to quantify the uptake of oxLDL using Oil Red O. As can be seen in Figure 7, oxLDL uptake was reduced in the presence of CD100 in all experiments, showing a suppressive effect in the LDL-induced foam cell differentiation.

### CD100 decreases ox LDL induced CD36 expression in macrophages

**Figure 8:**
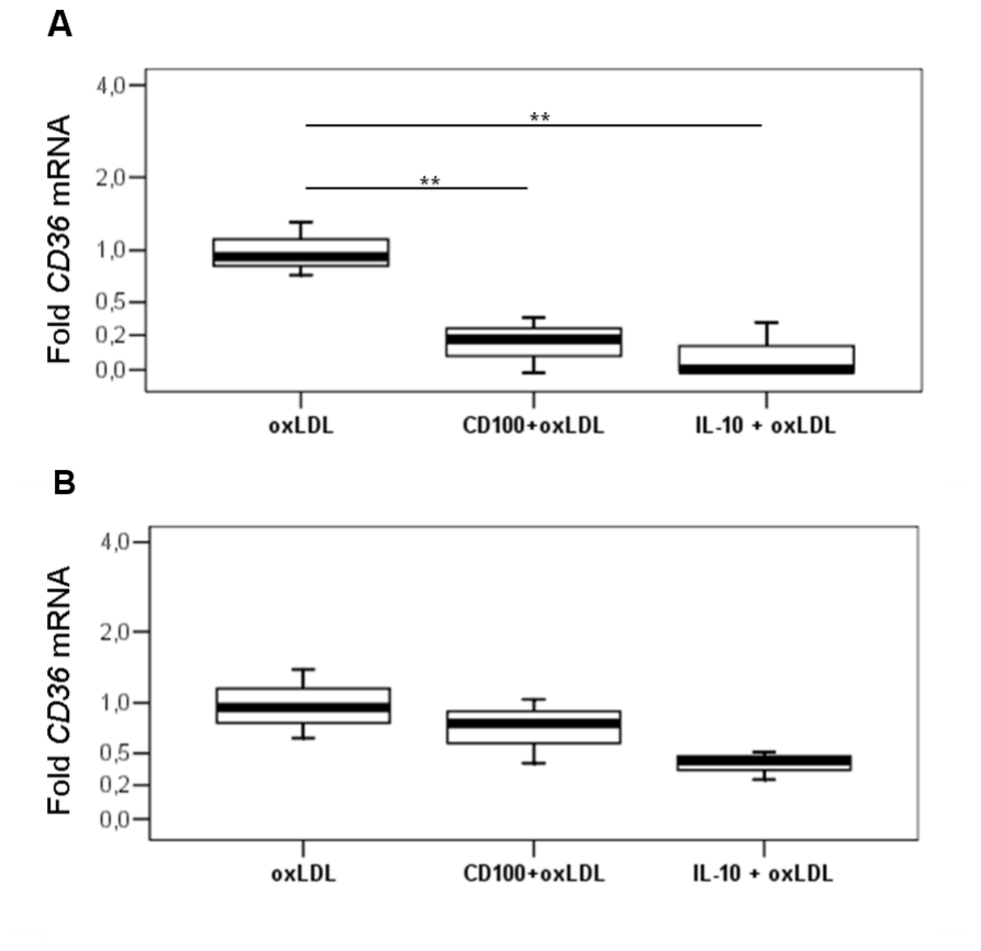
(A, B): CD36 mRNA is reduced in cultured macrophages treated with oxLDL in the presence of CD100 and IL-10. Relative quantitative RT-PCR (qRT-PCR) for CD36 in macrophages (**A**) and foam cells (**B**) incubated with oxLDL in the presence of CD100 or IL-10. Data normalized with GAPDH and expressed as a ratio (fold) relative to cells incubated only with oxLDL. Mean ± SD of 3 independent experiments (** p≤0.01).

To identify the mechanism by which CD100 down-regulates macrophage oxLDL uptake we analyzed CD36 mRNA expression (which is known to be induced by oxLDL) in these cells and in differentiated foam cells after CD100 treatment. Macrophages and foam cells were incubated with oxLDL with either CD100 or IL-10 (a CD36 down-regulator) (Figure 8A and B). As shown in Figure 8, CD100 and IL-10 significantly diminished CD36 expression in macrophages (Figure 8A) but not in foam cells (Figure 8B).

## Discussion

Phage display has proven to be an efficient tool for the identification of markers for several tissues and cell types [7–10]. The use of fresh human tissues (*ex vivo*) [28–31]or mouse models (*in vivo*) [7,8,28,32,33]for phage selection enhances the possibility of finding a peptide/antibody that can bind to human tissues *in vivo*, improving the diagnosis of the associated diseases. Such strategies have been successfully employed for tumors [28], and even for atherosclerosis [32,34].

In this work, phage technology indicated increased CD100 expression in fresh human carotid plaque samples. Despite the available studies in animal models, our study is the first to describe CD100 expression in human atherosclerotic plaques and to speculate a role for the molecule in the human disease. In fact, we observed remarkable CD100 expression in the lipid rich area of the plaques, particularly in macrophages and foam cells, whereas in the normal samples positive staining was observed only in endothelial cells. The isolation of a CD100 binding phage after subtraction using normal carotid (that express CD100 in endothelial cells) and panning of carotid plaques (that express CD100 mainly in macrophages and foam cells) may have been possible because macrophages and foam cells express more CD100 than endothelial ones (data not shown). This result is in line with previous data that show CD100 distinct activities in the immune system and its significant association in vascular phenomena.

CD100 had already been described in monocytes [14] and tumor associated macrophages [18], but not in other macrophages. CD100 positivity in splenic macrophages (Figure S1) suggested that the molecule is expressed by macrophages in general.

Monocytes, cultured macrophages and *in vitro* differentiated foam cells also showed positive staining for CD100, suggesting that the molecule participates in the different stages of the disease. Monocytes expressed the highest levels of CD100 RNA also after IFN-γ activation, suggesting important roles for the molecule in this cell type. Foam cells did not augment CD100 upon IFN-γ, and were also the least responsive cell type, as seen by STAT-1 levels. These results show that distinct cells respond differently to IFN-γ activation in terms of CD100 expression. Additionally, CD100 expression significantly diminished along monocyte-macrophage-foam cell differentiation, regardless of their activation state.

CD100 protein was detected in monocytes, macrophages and foam cells but showed no correlation to differentiation, contrarily to RNA data. Possible hypotheses for the lack of agreement between RNA and protein could be cleavage and shedding of the membrane CD100 by metalloproteases, later translation of CD100 transcripts than the time analyzed, or instability of CD100 transcripts, which would be more susceptible to degradation than the protein itself. Further experiments are needed to confirm one or more hypotheses.

Since the accumulation of lipid-laden macrophages is the first step of atherogenesis, we hypothesized whether CD100 could affect this process. CD100 treatment actually reduced LDL-induced foam cell formation. The mechanism by which this semaphorin reduces lipid uptake is probably by down-regulating the expression of CD36, a scavenger receptor with central role in oxLDL binding and atherogenesis. Despite CD36 importance in immune responses and vascular biology, there is surprisingly little knowledge regarding the regulation of its expression, and its modulation by CD100 was a novel finding. Further studies should be performed regarding possible uses of CD100 in anti-atherogenic therapies.

## Supporting Information

Figure S1 **CD100 is expressed by splenic macrophages.** Immunofluorescence of spleen (A-D), showing blue nuclei in DAPI (A), CD68 (green, B) and CD100 (red, C). The yellow color in the merged images (bottom right panel, D) denotes co-localization. Scale bars 20μm.
(TIF)
