# Supplementary figures and images for: Phage Display Identification of CD100 in Human Atherosclerotic Plaque Macrophages and Foam Cells
Source: PLoS One. 2013 Sep 30;8(9):e75772. doi: 10.1371/journal.pone.0075772 (PMC3787062; doi:10.1371/journal.pone.0075772)

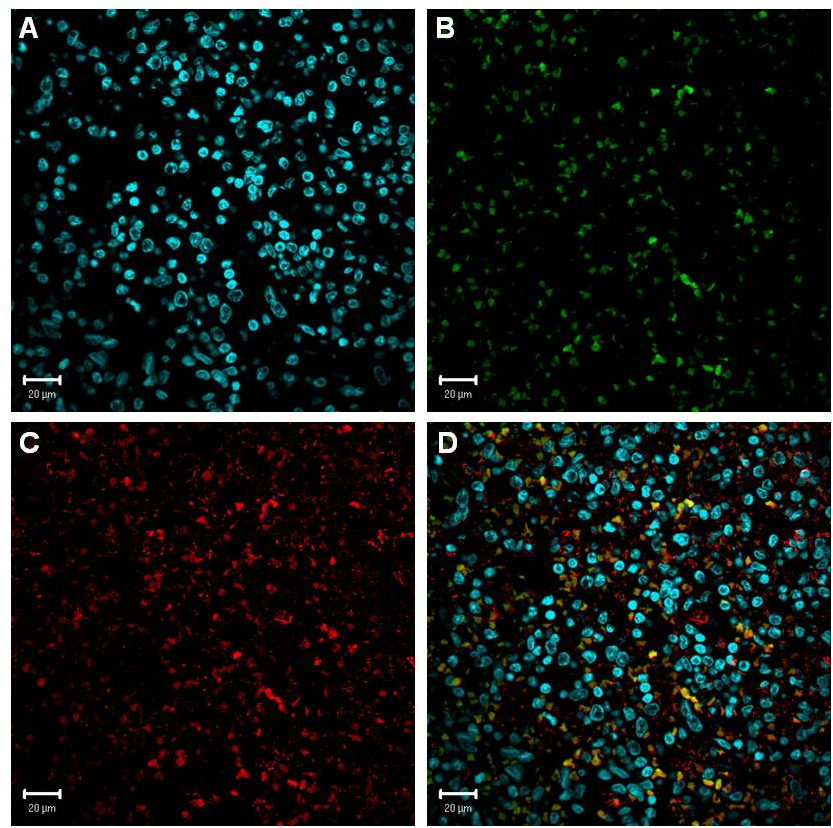

Supplement: Figure S1 — CD100 is expressed by splenic macrophages. Immunofluorescence of spleen (A-D), showing blue nuclei in DAPI (A), CD68 (green, B) and CD100 (red, C). The yellow color in the merged images (bottom right panel, D) denotes co-localization. Scale bars 20μm. (TIF) [file pone.0075772.s001.tif]
